# PHLPP Inhibitor NSC74429 Is Neuroprotective in Rodent Models of Cardiac Arrest and Traumatic Brain Injury

**DOI:** 10.3390/biom12101352

**Published:** 2022-09-23

**Authors:** Travis C. Jackson, Cameron Dezfulian, Vincent A. Vagni, Jason Stezoski, Keri Janesko-Feldman, Patrick M. Kochanek

**Affiliations:** 1 Department of Molecular Pharmacology & Physiology, Morsani College of Medicine, University of South Florida, 12901 Bruce B Downs BLDV, Tampa, FL 33612, USA; 2 USF Health Heart Institute, Morsani College of Medicine, University of South Florida, 560 Channelside Dr, Tampa, FL 33602, USA; 3 Safar Center for Resuscitation Research, UPMC Children’s Hospital of Pittsburgh, Rangos Research Center—6th Floor, Pittsburgh, PA 15224, USA; 4 Department of Pediatrics, Baylor College of Medicine, 6651 Main Street, Houston, TX 77030, USA; 5 Department of Critical Care Medicine, University of Pittsburgh School of Medicine, 4401 Penn Avenue, Pittsburgh, PA 15224, USA

**Keywords:** PHLPP, PHLPP1, PHLPP2, neuroprotection, NSC74429, brain

## Abstract

Pleckstrin homology domain and leucine rich repeat protein phosphatase (PHLPP) knockout mice have improved outcomes after a stroke, traumatic brain injury (TBI), and decreased maladaptive vascular remodeling following vascular injury. Thus, small-molecule PHLPP inhibitors have the potential to improve neurological outcomes in a variety of conditions. There is a paucity of data on the efficacy of the known experimental PHLPP inhibitors, and not all may be suited for targeting acute brain injury. Here, we assessed several PHLPP inhibitors not previously explored for neuroprotection (NSC13378, NSC25247, and NSC74429) that had favorable predicted chemistries for targeting the central nervous system (CNS). Neuronal culture studies in staurosporine (apoptosis), glutamate (excitotoxicity), and hydrogen peroxide (necrosis/oxidative stress) revealed that NSC74429 at micromolar concentrations was the most neuroprotective. Subsequent testing in a rat model of asphyxial cardiac arrest, and in a mouse model of severe TBI, showed that serial dosing of 1 mg/kg of NSC74429 over 3 days improved hippocampal survival in both models. Taken together, NSC74429 is neuroprotective across multiple insult mechanisms. Future pharmacokinetic and pharmacodynamic (PK/PD) studies are warranted to optimize dosing, and mechanistic studies are needed to determine the percentage of neuroprotection mediated by PHLPP1/2 inhibition, or potentially from the modulation of PHLPP-independent targets.

## 1. Introduction

Studies in mice show that knockout (KO) of the pleckstrin homology domain and leucine rich repeat protein phosphatase (PHLPP) gene improves CNS outcomes in mouse models of stroke, and traumatic brain injury (TBI), and decreases detrimental vascular remodeling after damage to the carotid artery [1,2,3]. PHLPPs promote cell death by inhibiting or activating a variety of signaling molecules. For instance, protein kinase B (AKT) is a potent pro-survival kinase and is inactivated by PHLPP-dependent dephosphorylation at Serine 473 [4]. Conversely, mammalian sterile 20-like kinase 1 (Mst1) is a potent pro-apoptotic effector and is activated by PHLPP-dependent dephosphorylation at Threonine 387 [5]. Thus, PHLPP antagonists, by blocking phosphatase activity, have great clinical potential to promote brain health by modulating neuronal survival and vascular homeostasis. Germane to that possibility, Sierecki et al. identified ~116 small molecule experimental PHLPP inhibitors [6]. All had IC50s ranging from 1–100 µM to inhibit the PHLPP2-PP2C phosphatase domain in a cell-free assay, and also blocked the PP2C domain of the PHLPP1 isoform with varying potencies [6]. Most of those inhibitors have not been characterized further and their research utility or translational potential is unknown. The exceptions are PHLPP inhibitors NSC117079 and NSC45586. Sierecki et al. showed that both compounds inhibited PHLPP signaling at ~50 µM in mammalian cells in vitro and were cytoprotective in a model of etoposide-induced cell death [6]. Consistent with that finding, we reported that 50 µM NSC117079 or NSC45586 altered PHLPP signaling in primary rat cortical neurons in vitro and were neuroprotective following staurosporine injury [7]. However, we also observed that NSC45586 strongly bound to albumin in the culture media, raising questions on its potential translatability for in vivo use [7]. In addition, both NSC117079 and NSC45586 are poor candidates to cross the blood-brain barrier (BBB) based on their physiochemical properties. Here, we used a predictive chemistry-guided approach to select three additional PHLPP inhibitors for testing that have not been further explored among the 116 known compounds, and to use three in vitro assays and two in vivo models to assess neuroprotection.

## 2. Materials and Methods

**Reagents.** Experimental PHLPP Inhibitors: NSC13378, NSC25247, and NSC74429 were obtained from the Developmental Therapeutics Program (DTP) of the National Cancer Institute (NCI) upon completion of a Material Transfer Agreement. Compounds were provided as powder in opaque glass vials and dissolved in 100% dimethylsulfoxide (DMSO) before diluting to the desired final concentration. DMSO, 30% Hydrogen Peroxide Solution, and Glutamate were purchased from Sigma (St. Louis, MO, USA). Staurosporine was purchased from Tocris (Bristol, UK). Cell Titer Blue was purchased from Promega (Madison, WI, USA). Neurobasal/B27 culture media was purchased from ThermoFisher Scientific/Life Technologies (Waltham, MA, USA).

**Animals.** All studies were approved by the IACUC of the University of Pittsburgh. Methods of euthanasia adhered with the AVMA Guidelines for the Euthanasia of Animals. Timed pregnant female Sprague Dawley rats were purchased from Charles River (Wilmington, MA, USA). Male adult Sprague Dawley rats (~12 weeks old) weighing 350–375 g were purchased from Charles River. Adult male C57BL/6 mice (~12 weeks old) weighing ~28 g were also purchased from Charles River. All animals were given ad lib access to food and water and housed on a 12 h light/dark cycle.

**Preparation of Experimental PHLPP Inhibitors for In Vitro and In Vivo Testing.** For in vitro studies, NSC13378, NSC25247, and NSC74429 were prepared in 10 mM stocks in 100% DMSO and diluted to a final concentration of 25 µM or 50 µM in cell culture media. For in vivo studies, pilot testing indicated low water solubility of NSC74429. Therefore, a combination of DMSO, heating via a water bath, and vigorous vortexing was used to enhance solubilization. Two drug doses were tested in rats and in mice (1 or 3 mg/kg). For studies in rats, NSC74429 was prepared in 24% DMSO/D5W (dextrose 5% in water) and administered at a volume of 1.25 mL/kg (intravenous [IV] or intraperitoneal [IP]). For studies in mice, NSC74429 was prepared in 5% DMSO/D5W (vehicle) and a 250 µL bolus was administered IV or IP.

**Primary Neuronal Culture.** Neuron culture was done using our established methods [7,8]. In brief, timed-pregnant Sprague Dawley rats were purchased from Charles River and cortices isolated from E18-19 embryos under a dissecting microscope (Leica M651, Buffalo Grove, IL, USA). Mixed-sex neurons were dissociated and seeded onto 96-well plates at 1.5 × 10^5^/well in Neurobasal/B27 media. The culture media was supplemented with 8 µM cytosine β-D-arabinofuranoside hydrochloride on day in vitro (DIV) 3 to reduce glial proliferation. A second feeding (½ media exchange) was given on DIV6 and neurons were subjected to injury and/or drug therapy on DIV9.

**Staurosporine Injury Model.** On DIV9, half of the media from each well was collected and pooled (conditioned media) and mixed with an equal volume fresh neurobasal/B27 to make “24 h treatment media”. Treatment media was prepared with or without 150 nM STS and with or without experimental PHLPP inhibitors at 25 µM or 50 µM. The final concentration of DMSO in all groups was ~0.5%. Each well received 100 µL of treatment media per well and incubated for 24 h at 37 °C. Cell viability was measured on DIV10.

**Hydrogen Peroxide (H_2_O_2_) Injury Model.** On DIV9, 2/3 conditioned media was prepared and set aside for the post-injury phase. H_2_O_2_ was prepared fresh. Specifically, 30% concentrated H_2_O_2_ with stabilizers (Sigma) was first diluted in sterile ddH_2_O. The diluted H_2_O_2_ stock (Stock A) was immediately added to a balanced salt solution containing 5 mM glucose (BSS-G) and diluted to a final concentration of 40 µM H_2_O_2_ (Stock B). Cells were quickly washed once with plain BSS-G and then incubated with 40 µM H_2_O_2_ for 35 min. Controls received identical manipulations without injury. After 35 min, the Stock B injury solution was removed and 100 µL of conditioned media with or without experimental PHLPP inhibitors was added to the cells. The final DMSO concentration was ~0.5%.

**Glutamate-Excitotoxicity Model.** On DIV9, 2/3 conditioned media was prepared and set aside for the post-injury phase. Cells were quickly washed once with plain BSS-G and then incubated in BSS-G containing 10 µM Glutamate for 5 min. Controls received identical manipulations without injury. After 5 min, the glutamate solution was removed and 100 µL of conditioned media with or without experimental PHLPP inhibitors was added to the cells. The final DMSO concentration was ~0.5%.

**Cell Viability Assay.** In vitro neuronal injury was assessed using the CellTiter-Blue method as reported by our group [7,8]. Briefly, 20 µL of CellTiter-Blue reagent (Promega; Madison, WI, USA) was added to each well of a 96-well plate at 24 h post-injury. Plates were incubated in the dark for ~1 h and absorbance was measured on a plate reader.

**Asphyxial Cardiac Arrest Rat Model.** Rats were injured by ACA as described by our group [9]. Rats were fasted overnight prior to surgery. Anesthetized rats (4% isoflurane in a 30:70 mix of oxygen/nitrogen) were intubated and mechanically ventilated (~45–50 breaths/min). Positive end-expiratory pressure (PEEP) was set to 3 cm H_2_O and tidal volume to 7 mL/kg. Temperature was monitored with a rectal thermometer and a tympanic probe (Physitemp Instruments LLC, Clifton, New Jersey, USA) and adjusted via a heating pad/lamp to target ~36.5–37 °C. Electrocardiographic leads were attached to the limbs for continuous ECG monitoring (Powerlab, ADinstruments, CO, USA). Femoral artery and venous catheters (PE50) were surgically inserted in the left hind limb for continuous blood pressure/heart rate monitoring (arterial) and for drug injections (venous). Ventilator rate, tidal volume and FiO2 were adjusted based on arterial blood gas data to achieve goal PaCO2 of 35–40 mm Hg and PaO2 of 80–150 mm Hg. To induce ACA, isoflurane was discontinued just prior to cisatracurium (2 mg/kg) administration to induce neuromuscular blockade, and rats were then disconnected from the mechanical ventilator, “No-flow” was considered achieved when mean arterial blood pressure (MAP) reached ≤10 mmHg and was maintained for 5 min in all subjects prior to starting resuscitation efforts. The total asphyxia insult time was ~8–9 min and = (a) the time of the hypoxic period starting from the discontinuation of ventilation until no-flow is reached + (b) 5 min no-flow time + (c) the time to achieve return of spontaneous circulation (ROSC) after starting resuscitation maneuvers. Subjects that were not able to be resuscitated within 2 min of the onset of chest compressions were out of protocol and excluded from the study. Resuscitation was achieved by reinitiating mechanical ventilation, starting mechanical chest compressions using a rat thumper at a rate of 275 beats per minute, and IV administration of epinephrine (20 µg/kg), and sodium bicarbonate (1 mg/kg). At 5 min post-restoration of spontaneous circulation (ROSC) rats received vehicle or NSC74429 (1 or 3 mg/kg) IV, and booster doses were administered via IP injections at 1 d, 2 d, and 3 d post-ROSC. At 15 min post-ROSC, rats received 5 mg/kg of ketoprofen to treat surgical pain, and booster IP injections followed deficit scoring (5 mg/kg ketoprofen) on days 1, 2, and 3 post-injury. A MiniMitter (Philips Respironics, Murrysville, PA, USA) temperature probe was inserted into the abdominal cavity for telemetric monitoring of temperature to ensure normothermia for the first ~18 h of recovery. Femoral catheters were removed, wounds sutured, and rats received 10 mL subcutaneous Dextrose 5% (D5NS) to ensure hydration. Outcome performance category (OPC; 1 = normal, 2 = mild disability, 3 = moderate disability, 4 = severe disability, and 5 = death) and neurological deficit score (NDS; 0–500, 0 = normal and 500 = brain dead) were assessed daily for the first 72 h and on 7 d as described by our group [9,10]. After 72 h, rats were returned to the primary animal care facility until euthanasia for histological endpoints on day 7. Shams received surgical manipulations and vehicle injections. Rats were randomized to treatments and technicians were blind to groups (e.g., de-identified tubes labeled “A”, “B”, or “C” were provided to the technician that administered the experimental therapy after ACA injury and for subsequent booster doses). Rats that died before reaching the 7 d endpoint were removed from statistical analysis. Three rats failed to achieve ROSC (death before drug therapy), and 4 were excluded for death after ROSC but before day 7 (1 vehicle, 1 low dose NSC74429, 2 high dose NSC74429).

**Traumatic Brain Injury Mouse Model.** Mice were injured by controlled cortical impact (CCI) followed by a secondary hemorrhagic shock (HS) insult (to mimic the commonly observed clinical polytrauma scenario) as described by our group [11,12]. In brief, anesthetized mice (4% isoflurane in 70% N_2_O/30% O_2_) were secured inside a stereotaxic frame. Body temperature was recorded via a rectal probe (targeted to ~37 °C), and catheters inserted into the left femoral artery and vein. A dental drill was used to perform a craniotomy over the left parietal cortex. A pressure-driven pneumatic impactor, with a 3 mm steel flat tip, was advanced into the brain parenchyma (velocity 5/ms; depth 1.0 mm). Five minutes after the CCI, isoflurane was reduced to 0.5%, and blood was removed by the femoral venous catheter connected to a syringe containing citrate anticoagulant. Blood was withdrawn over 15 minutes until MAP reached 25 to 27 mmHg. Hemorrhagic shock was pressure-controlled for 35 mins by removing or administering shed blood in 50 μL aliquots. After HS, volume resuscitation was initiated with 20 mL/kg lactated ringers (LR) solution, plus additional bolus injections of 10 mL/kg LR as needed to sustain a target MABP of 70 mmHg for 90 min (Pre-Hospital Care Phase). The final ‘Hospital Phase’ was initiated by returning shed blood to mice over 15 mins. After re-infusion of the shed blood, MAP generally returns to near baseline levels (~90 mm Hg). Immediately following the Hospital Phase, mice were administered the experimental therapy IV (vehicle, 1 mg/kg, or 3 mg/kg). Mice were weaned from isoflurane, catheters were removed, and they were allowed to recover on supplemental oxygen for 30 mins before returning to the animal housing facility. In the acute study cohort (48 h endpoint) mice received an additional IP bolus injection of the experimental therapy at 24 h post-injury. In the subacute study cohort (7 d endpoint) mice received additional IP bolus injections at 1 d, 2 d, and 3 d post-injury. Shams received craniotomy, catheterization, and vehicle injections. Mice were randomized to treatments and technicians were blind to groups (e.g., de-identified tubes labeled “A”, “B”, or “C” were provided to the technician that administered the experimental therapy after CCI + HS injury and for subsequent booster injections).

**Hematoxylin and Eosin (H&E) Staining and Cell Counts.** Histological staining and analysis were done as described by our group [2]. In brief, at the determined study endpoint, rodents were anesthetized and flushed with heparinized ice-cold saline by transcardial perfusion into the left ventricle with drainage from a right atrial incision until all blood was cleared. Subsequently, the animal was immediately flushed with 10% buffered formalin. Brains were removed and postfixed in 10% buffered formalin for 72 h. Brains were cut, dehydrated, cleared, embedded into paraffin wax, and sectioned on a microtome. Sections were deparaffinized, treated with hematoxylin stain (ThermoScientific, Waltham, MA, USA), washed, treated with Bluing reagent (ThermoScientific), washed, and treated with Eosin Y (ThermoScientific), mounted with a coverslip on glass slides, and imaged on a Nikon Eclipse 90i microscope (Nikon, Melville, NY, USA). Live cells (CA1 and CA3) in the stratum pyramidale hippocampus were counted, divided by the length of the CA1/CA3, and reported as cells/0.1 mm. The technician quantifying CA1/CA3 cell counts was blind to treatment.

**Statistics.** Fluorescent units (CellTiter-Blue viability) were analyzed by one-way ANOVA followed by a post hoc Newman-Keuls multiple comparison adjustment. CA1 cell counts from ACA injured rats were analyzed by one-way ANOVA followed by Dunnett’s post hoc comparison with vehicle-injury established as the control group for group comparisons. The CA1 cell count for a single subject was eliminated from statistical analysis because it was a statistical outlier as determined by the ROUT method. No other subjects across all groups were identified as outliers in the ROUT analysis. The one outlier animal received a ~9 min cardiac arrest in the vehicle treated group but had the highest CA1 cell count across all groups including when compared to uninjured sham rats. OPC and NDS data were analyzed by two-way ANOVA followed by Dunnett’s post hoc comparison with vehicle-injury established as the control group for group comparisons. In the 48 h CCI + HS study, cell count data between groups had unequal variance. Therefore, 48 h CA1/CA3 cell count data were analyzed by Welch’s ANOVA followed by Dunnett’s T3 post hoc comparison test with vehicle-injury established as the control group for group comparisons. For the 7 d CCI + HS cohort, descriptive statistics confirmed all groups had equal variance. Therefore, a standard two-way ANOVA followed by Dunnett’s post hoc comparison was performed, with vehicle-injury established as the control group for group comparisons. Scatter plots were graphed in GraphPad Prism (GraphPad Software Inc., La Jolla, CA, USA, Version 8.1.2). Data were significant at *p* < 0.05.

## 3. Results

**Figure 1:**
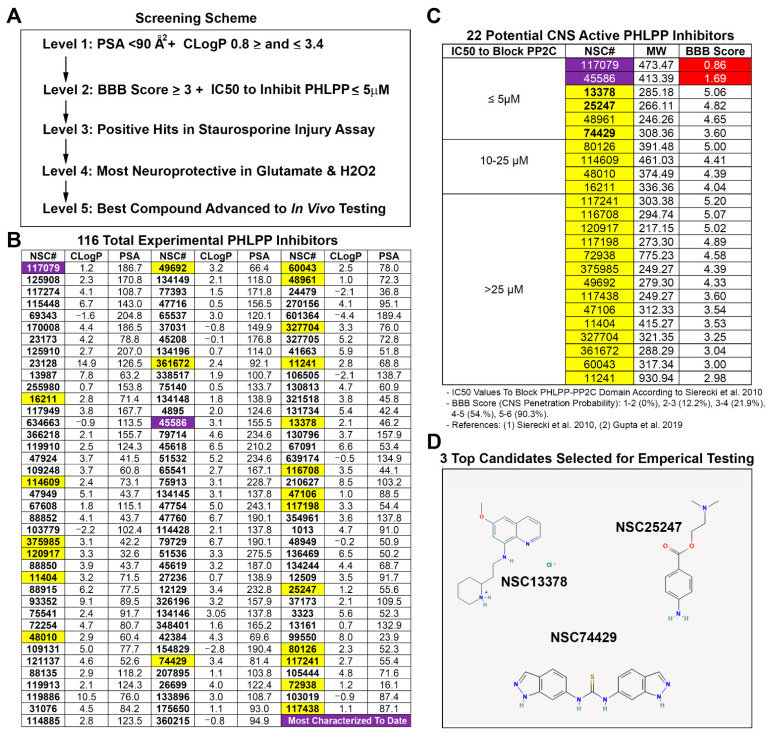
Physicochemical Properties of PHLPP Inhibitors and the Prediction of Their CNS Activity. (**A**) An overview of the top-down screening process employed in this study for drug selection. (**B**) The predicted polar surface aera (PSA) and calculated partition coefficient (CLogP) for each PHLPP inhibitor calculated in Chemicalize (Chemaxon). Compounds highlighted in purple denote the two PHLPP inhibitors that have received the most empirical study/characterization to date. Compounds highlighted in yellow denote unexplored PHLPP inhibitors that have chemistries supportive of BBB penetration. (**C**) The 2 most studied compounds and the 22 unexplored PHLPP inhibitors organized by known IC50s to block the PP2C domain of PHLPP2. Additionally, the BBB Score (BBB-S) was calculated for each of the compounds and arranged in each subgroup highest to lowest. The red highlighted BBB Scores indicate values < 2 and consistent with non-CNS drugs [6,18]. (**D**) The chemical structures of 3 unexplored PHLPP inhibitors chosen for testing in neurons is depicted.

A five-step screening strategy (Figure 1A) was used to identify a potential CNS permeable PHLPP inhibitor for neuroprotection. Polar surface area (PSA) and calculated partition coefficient (CLogP) are critical chemical properties that influence BBB penetration of compounds [13,14]. PSA and CLogP were calculated for each of the 116 known PHLPP inhibitors using Chemicalize software (Chemaxon; Boston, MA, USA) (Figure 1B). The upper limit of the PSA for BBB penetrating compounds is <90 Å2 [13]. This led us to eliminate 65 PHLPP inhibitors from further consideration. The remaining 51/116 compounds were screened for CLogP. The optimal mean CLogP of BBB penetrating drugs was reported to be 2.1 [13,15], 2.5 [13,16], or 3.4 [13,17]. However, CLogP of BBB penetrating drugs is normally distributed on a bell curve ranging from ~1 to ~5 [14]. To support our goal of refining the number of hits, we set 2.1 as the optimal CLogP then instituted cutoffs by adding or subtracting 1.3 from that value (i.e., a minimum of 0.8 and a maximum of 3.4). Thus, we selected for compounds that cluster near the optimal CLogP mean of 2.1–3.4 and which led us to eliminate an additional 29 inhibitors from further consideration.

To further rank the 22 remaining PHLPP inhibitors, we calculated the BBB Score for each compound. The BBB Score methodology developed by Gupta et al. [18] considers five physicochemical descriptors including: (a) number of aromatic rings, (b) heavy atoms, (c) a descriptor comprising molecular weight, hydrogen bond donor, and hydrogen bond acceptors (i.e., the MWHBN), (d) the PSA, and (e) pKa. The utility of the BBB Score was verified by Gupta et al. against 270 CNS active and 720 non-CNS FDA-approved drugs [18]. In their study no compounds with a calculated BBB Score of ≤2 were CNS active. A total of 12.8% of drugs with a BBB Score between 2 and 3 were CNS active; 21.9% of drugs with a BBB Score between 3 and 4 were CNS active; 54.5% of drugs with a BBB Score between 4 and 5 were CNS active; and 90.3% of drugs with a BBB Score between 5 and 6 were CNS active. Germane to the 22 PHLPP inhibitors deemed of interest, 21/22 had BBB Scores ≥ 3, 14/22 had scores > 4, and 4/22 had scores ≥ 5. (Figure 1C). Notably, the two most prolifically investigated PHLPP inhibitors have a BBB score < 2, and the frontrunner for potential translation (NSC177079 [6,7,19,20]) had the lowest value of 0.86. The 22 unexplored inhibitors were then grouped and ordered by IC50 to block the PP2C domain of PHLPPs [6]. Only compounds with the lowest IC50 to inhibit PHLPP (≤5 µM) were considered for empirical testing of neuroprotection. This led us to consider four compounds for in vitro studies (NSC13378, NSC25247, NSC48961, and NSC74429), Ultimately, NSC48961 was excluded due to having a CLogP furthest outside the optimal range (2.1–3.4) and because better options were identified. This led us to obtain from the DTP/NCI three highly promising compounds to test in cell culture (Figure 1D): NSC13378 (BBB Score = 5.06), NSC25247 (BBB Score = 4.82), and NSC74429 (BBB Score = 3.60).

**Figure 2:**
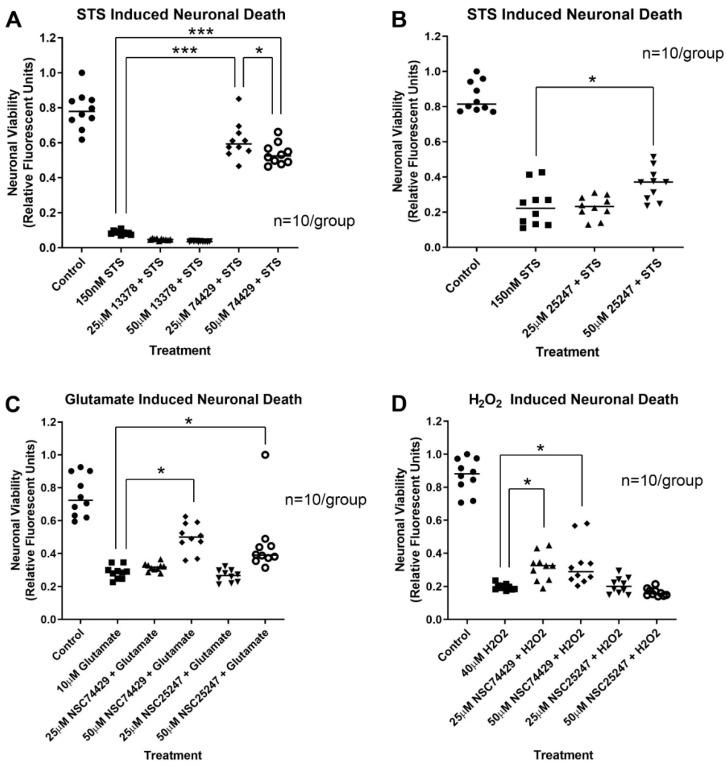
Neuroprotective Efficacy of NSC13378, NSC25247, and NSC74429 in Cultured Primary Rat Cortical Neurons. Neurons were injured on day in vitro (DIV) 9 and treated with vehicle or NSC compounds. (**A**) A scatter plot showing 24 h viability in staurosporine injured neurons treated with NSC13378 or NSC74429. (**B**) A scatter plot showing 24 h viability in staurosporine injured neurons treated with NSC25247. (**C**) A scatter plot showing 24 h viability in glutamate injured neurons treated with NSC25247 or NSC74429. (**D**) A scatter plot showing 24 h viability in hydrogen peroxide injured neurons treated with NSC25247 or NSC74429. All groups include n = 10/group. Data were analyzed by ANOVA and post hoc significance was detected using the Newman-Keuls multiple comparison test. Data were significant at *p* < 0.05. (*) = *p* < 0.05, (***) = *p* < 0.001.

NSC13378 did not increase neuronal survival in primary rat cortical neurons after a 24 h staurosporine insult (Figure 2A). In contrast, NSC25247 (at 50 µM) and NSC74429 (at 25 and 50 µM) were both neuroprotective in the STS assay (Figure 2A,B). NSC13378 was eliminated from further consideration while the other two compounds were advanced to testing in models of excitotoxicity and oxidative stress. NSC25247 increased 24 h neuronal survival after glutamate-induced injury (at 50 µM) but had no effect on survival in the H_2_O_2_ injury assay (Figure 2C,D). In contrast, NSC74429 increased 24 h neuronal survival in both the glutamate (at 50 µM) and H_2_O_2_ injury assay (at 25 and 50 µM) (Figure 2C,D). At the highest dose tested NSC25247 was neuroprotective in 2/3 assays and was a promising candidate for a CNS-active PHLPP inhibitor. However, it was eliminated from further consideration because NSC74429 demonstrated superior characteristics (i.e., it protected in 3/3 assays at one or both concentrations).

**Figure 3:**
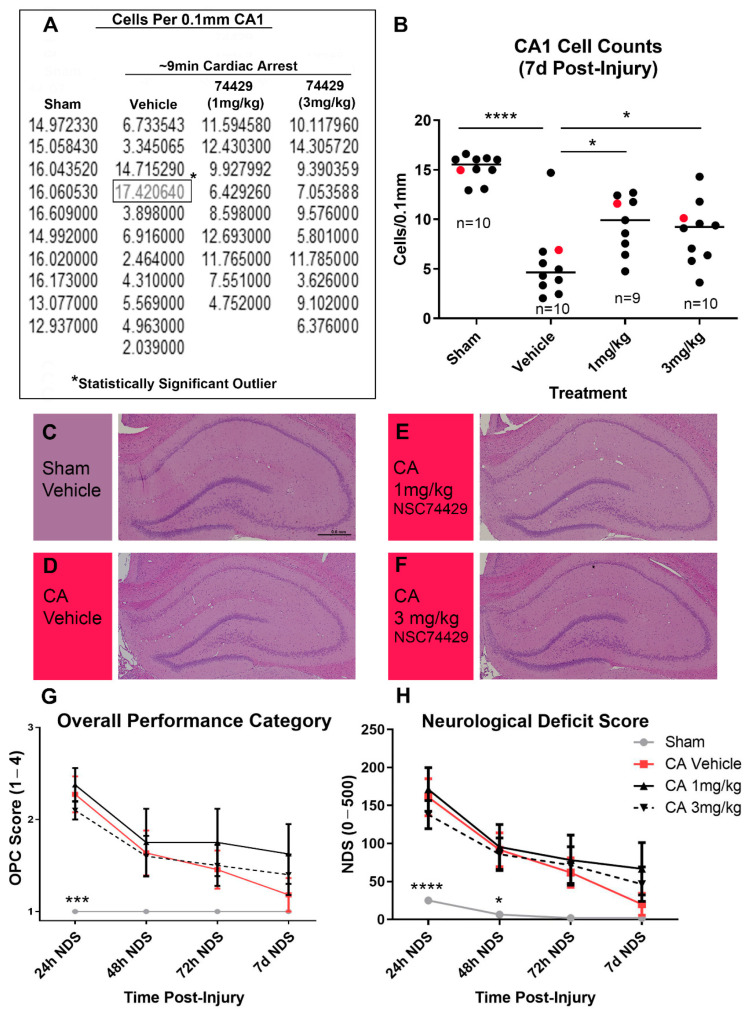
Evaluation of NSC74429 Mediated Neuroprotection in a Rat Model of Cardiac Arrest. (**A**,**B**) Normalized CA1 cell count values are shown along with the associated scatter plot. A single CA1 cell count outlier was confirmed by the ROUT method (indicated by the *) and removed prior to statistical analysis of histology. Individual data points highlighted in red in the scatter plot correspond to the rat brains selected as representative images. (**C**–**F**) Representative images of H&E-stained brains used to assess CA1 cell loss. (**G**,**H**) Line graphs (mean + SEM) show changes in overall performance category (OPC) and neurological deficit score (NDS) testing measured in the first 72 h post-injury and at 7 d post-injury. Asterisks indicate significance compared to vehicle-injured rats. Data were significant at *p* < 0.05. (*) = *p* < 0.05, (***) = *p* < 0.001, (****) = *p* < 0.0001.

Rats underwent an ~8–9 min ACA and were administered post-insult vehicle, 1 mg/kg NSC74429, or 3 mg/kg NSC74429. Both doses of the experimental PHLPP inhibitor increased 7 d neuronal survival in the CA1 subregion of the hippocampus (Figure 3A–F and Figure S1). The duration of the total ischemia time did not differ compared to vehicle (IQR [min:sec]: vehicle 8:39–9:07, 1 mg/kg 8:34–8:56, 3 mg/kg 8:39–9:02; post hoc Dunnett’s *p* = 0.856 for vehicle vs. 1 mg/kg and *p* = 0.982 for vehicle vs. 3 mg/kg), indicating that differences in 7 d neuron survival were not due to differences in insult severity. OPC and NDS testing did not reveal significant differences in neurological recovery at 1 d, 2 d, 3 d, or 7 d post-injury in vehicle versus NSC74429 treated rats (Figure 3G,H).

**Figure 4:**
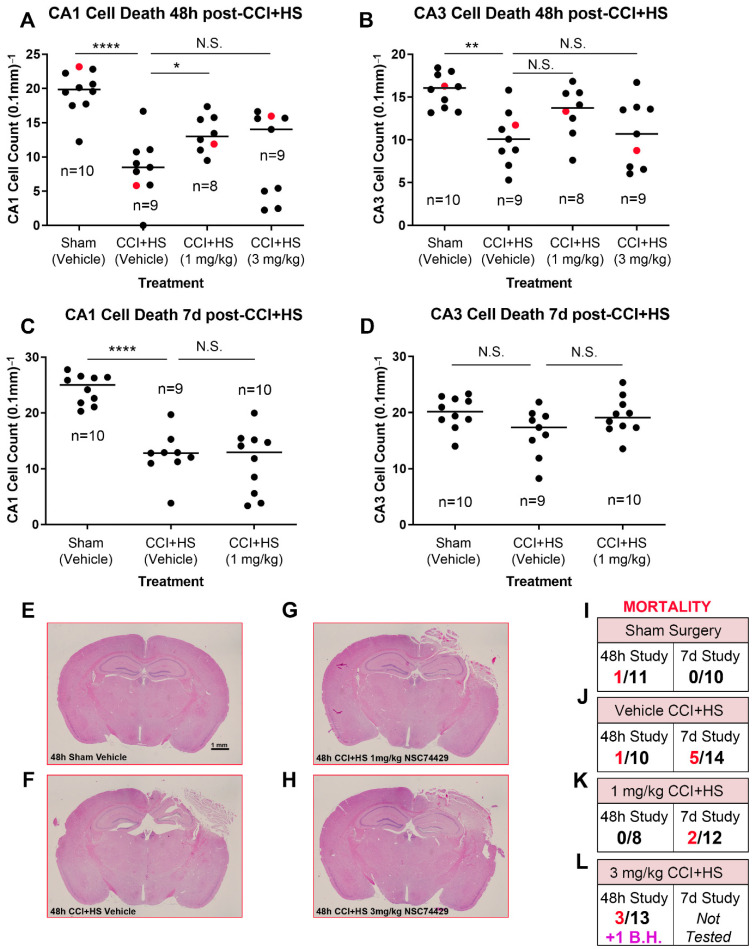
Evaluation of NSC74429 Mediated Neuroprotection in a Mouse Model of TBI. (**A**,**B**) Scatter plots of normalized CA1 and CA3 cell counts at 48 h post-injury. (**C**,**D**) Scatter plots of normalized CA1 and CA3 cell counts at 7 d post-injury. Individual data points highlighted in red in the scatter plot correspond to the mouse brains selected as representative images in panels E-H. (**E**–**H**) Representative images of H&E-stained brains used to assess CA1/CA3 cell loss. (**I**–**L**) Mortality for each group by treatment and study endpoint. Asterisks indicate significance compared to vehicle-injured rats. The red text indicates animals that died. The purple text indicates an animal that survived to the study endpoint but had bleeding in the brain that precluded cell counting. Data were significant at *p* < 0.05. Not Significant (N.S.), Brain Hemorrhage (B.H.), Controlled Cortical Impact (CCI), Hemorrhagic Shock (HS). (*) = *p* < 0.05, (**) = *p* < 0.01, (****) = *p* < 0.0001.

Finally, to begin to assess the scope of hippocampal neuroprotection in vivo, we also tested NSC74429 in an established mouse model of severe TBI. Vehicle treated mice subjected to a CCI + HS had significant CA1 and CA3 neuronal loss in the hippocampus at 48 h post-injury (Figure 4A,B). By 7 d post-injury, neuronal loss persisted in the CA1 subregion but was not statistically different versus shams in the CA3 subregion (Figure 4C,D). One mg/kg NSC74429 significantly increased CA1 neuronal survival at 48 h post-injury but not at 7 d post-injury (Figure 4A,C). Additionally, 1 mg/kg NSC74429 was associated with a trend toward improved CA3 cell counts at both the 48 h and 7 d post-injury time points but this was not statistically significant (Figure 4B,D). CA1/CA3 neuronal survival in the 3 mg/kg treatment group was also not statistically different compared to vehicle treated mice (Figure 4A,B). However, CA1 cell counts at 48 h post-injury displayed a biomodal pattern in the high dose group—one subgroup appeared to show signs of neuroprotection, whereas the other subgroup appeared worse than vehicle treated mice (Figure 4A). Mortality was highest overall in the 3 mg/kg group (in the 48 h study) and had the only case of a surviving mouse with brain hemorrhage that prevented accurate hippocampal neuronal counting (Figure 4I–L). For that reason, 3 mg/kg was not further explored in the 7 d outcome study.

## 4. Discussion

Sierecki et al. used a combination of cell-free screening assays and in silico tools to identify ~116 small-molecules that selectively block the PP2C-phosphatase domain in PHLPPs [6]. These compounds are non-selective for PHLPP1 and PHLPP2. Two compounds (NSC117079 and NSC45586) were subsequently investigated by others but are unlikely candidates for CNS therapeutics [7,19,20]. Specifically, their chemical properties are not ideal for targeting the brain (e.g., their BBB score < 2) and some bind albumin.

Our objective was to employ an accelerated win/kill approach to quickly filter/identify, among the 114 unexplored PHLPP inhibitors, a single compound that merited in vivo testing for neuroprotection in multiple models of brain injury. Our rationale was based on the need for a PHLPP inhibitor that is more likely to target the brain, and to establish neuroprotection before advancing with elaborate follow up studies to comprehensively assess cell signaling in a variety of cell types, behavioral outcomes, pharmacokinetic and pharmacodynamic (PK/PD) studies, and brain disposition studies to determine dosing and clearance kinetics.

A five-staged algorithm was used to identify a promising PHLPP inhibitor candidate for CNS drug development (Figure 1A). First, we reviewed both key traditional chemical criteria (e.g., CLogP thresholds for CNS drugs [13]) and contemporary tools (e.g., calculation of the BBB Score [18]) to identify 22 compounds with chemical properties suggestive of BBB penetration (1st and 2nd level analysis). Next, we selected the top three drugs (NSC13378, NSC25247, and NSC74429) with optimal properties and the lowest IC50s to inhibit PHLPPs, and empirically tested them in neurons in a gold-standard model of STS-induced apoptosis (3rd level analysis). STS was the initial screening injury-assay because it models a pure apoptotic insult, and because apoptosis is the prototypical cell death pathway augmented by PHLPPs [4,21]. Furthermore, we previously reported that PHLPP gene knockdown or treating cells with NSC117079 is neuroprotective in STS, and therefore using the same assay here facilitated historical comparisons germane to the efficacy of the uncharacterized novel PHLPP inhibitors that were tested [7]. The most promising compounds advanced to testing in in vitro models of glutamate-induced excitotoxicity and H_2_O_2_-induced oxidative stress (4th level analysis). The insults produced by these agents activate a complex constellation of cell death mechanisms including apoptosis, necrosis, and autophagy [22,23,24,25]. These mechanisms are felt to be critical in the pathophysiology of clinical brain injury including in TBI, stroke and ACA [26,27,28]. NSC74429 performed best overall (on all three assays) and was advanced to animal testing (5th level analysis). We used a 1 mg/kg (low) and 3 mg/kg (high) dose for in vivo studies. Dosing was based on a similar dose range reported with NSC117079 and NSC45586 to induce a biological effect in animals [20]. To further strengthen our in vivo studies, we used two different brain injury models across two different species, to assess the generalizability and robustness of neuroprotection. Our rat ACA model results in a primary insult to the CA1 subregion of the hippocampus but does not damage the BBB [29,30]. In contrast, our mouse CCI + HS model is comparatively more severe, results in widespread necrosis, a cortical contusion, robust hippocampal CA1/CA3 neuron death, and the BBB is compromised as determined by Evans blue extravasation [11,31].

Low and/or high dose NSC74429 increased rat neuron survival after STS, glutamate, or H_2_O_2_ -induced cell death. High dose (50 µM) NSC25247 also showed promise and was neuroprotective in both the STS and glutamate injury models, but not in H_2_O_2_. Importantly, all assays employed a post-treatment strategy, except for STS in which NSC compounds were added at the same time as the injury agent. Additionally, the finding that low dose (25 µM) NSC74429 robustly protected in STS (apoptosis), modestly in H_2_O_2_ (necrosis), and had no effect in glutamate (excitotoxicity), precluded the possibility that neuroprotection was simply due to an artifact of the compound interacting with the viability assay. Similarly, cells treated with NSC13378 were visibly dead.

NSC74429 also decreased neuronal death in vivo in a rat model of ACA and a mouse model of TBI. Neuroprotection in the injured CA1 in the ACA model persisted for at least 7 d post-injury, which suggests that NSC74429 did not simply delay inevitable cell death—although even longer endpoints were not studied [32,33]. Additionally, the fact that BBB function is maintained in the ACA model, and that neuroprotection with NSC74429 was observed, supports the predictive chemistry suggesting that it is a promising candidate to target the brain. Nevertheless, future studies are needed to directly assess drug levels in brain tissue and/or to ascertain if peripheral effects could have contributed to CA1 neuroprotection via indirect mechanisms. In the CCI + HS model, CA1 neuroprotection was seen at 48 h but not 7 d post-injury. Thus, NSC74429 delayed cell death in CA1 in the TBI model but did not produce long-lasting neuroprotection in that region. The neuroprotective benefit on 48 h CA1 survival is nevertheless striking given that (a) the CCI + HS model produces a very severe insult which represents a high bar for neuroprotectants to show a benefit, and (b) the administration of NSC74429 was delayed by almost ~2 h after the initial CCI-TBI component of the insult (i.e., therapy was initiated after the hospital phase of the HS component). Interestingly, in the CA3, there was a trend toward a sustained increase in neuronal survival in the 1 mg/kg NSC74429 group at 48 h and 7 d post-injury; however, this effect was not significant, and our study was not powered to appropriately test that hypothesis. Higher mortality in the 3 mg/kg group merits additional study. While bolus dosing was a logical first step to assess neuroprotection, future PK/PD studies are needed to determine if sustained delivery at targeted serum doses may further improve histological outcomes and minimize adverse side effects. The mechanism(s) mediating increased mortality at the high bolus dose are unclear but will need to be elucidated prior to clinical translation, if warranted, or to potentially determine if modifications to the parent compound may mitigate some unwanted side effects.

We were surprised that NSC13378 failed to protect; it was our frontrunner based on its LogP, BBB Score, and IC50 to inhibit PHLPP. This unexpected result highlights the benefits of our multi-staged approach to screening. Conversely, we were surprised by the robustness of NSC74429. Indeed, the magnitude of neuroprotection appeared to be much greater than what we previously observed in the STS assay in primary rat neurons after PHLPP1 gene knockdown or by using NSC117079 [7]. Future cell culture studies are needed to test if protection with NSC74429 is lost or ameliorated after PHLPP1 or PHLPP2 knockdown, or after combined PHLPP1/PHLPP2 knockdown. Those experiments may shed light on the potential contribution of PHLPP-dependent versus -independent mechanisms mediating neuroprotection.

It is possible that NSC13378 failed to protect due to toxic off-target effects, whereas NSC74429 was beneficial due to neuroprotective off-target effects. Thus, it is not clear if hippocampal protection was fully or partially dependent on PHLPP inhibition and the specificity of NSC74429 remains to be determined. Importantly, contemporary thinking in neurotrauma has moved away from focusing solely on drugs that target a single mechanism. That is because the nature of brain damage seen in heterogenous TBI patients is multifactorial and has contributed, in part, to the lack of new interventions after >191 clinical trials [34]. Thus, from a translational perspective, we think it would be advantageous if future studies discover that NSC74429 targets additional neuroprotective mechanisms on top of PHLPP inhibition. We recognize however a disadvantage is that the more pathways targeted by NSC74429 (if confirmed) the less useful this compound may turn out to be as a research tool to selectively probe PHLPP biology in the brain, but whether this concern is justified remains to be seen. Additionally, it is equally possible that NSC74429 is a selective PHLPP inhibitor and that it performed the best because of its high specificity and lack of side effects at the doses tested.

Clinically, we think that our findings may be important. Pro-death PHLPP1 and PHLPP2, also referred to as suprachiasmatic nucleus circadian oscillatory proteins (SCOP), are encoded by two genes [4,35,36]. The PHLPP-PP2C domain is highly homologous between isoforms (58% identity), inhibits a variety of pro-survival signaling pathways, and is of interest germane to blocking PHLPP-PP2C activity for neuroprotection [1,36]. Indeed, PHLPP1 gene KO mice have decreased infarct volume after experimental stroke [1]. Additionally, PHLPP1 impairs hippocampal-dependent memory function, and gene KO mice have enhanced memory performance following a TBI [2,37]. PHLPP2 similarly promotes neuronal death and siRNA mediated gene knockdown decreased brain damage in a rat model of global cerebral ischemia [38]. More recently it was discovered that endothelium-specific PHLPP2 KO mice have decreased neointima formation in injured carotid arteries (i.e., decreased harmful vascular remodeling) [3]. Conversely, PHLPP2 levels are increased in the endothelium of human patients with atherosclerotic plaques [3]. Thus, PHLPPs are linked to a spectrum of disease-promoting cell signaling perturbations that could negatively impact chronic brain health, and pharmacological inhibitors targeting PHLPPs in the human brain may be useful.

Study limitations include sample size, the lack of an assessment of drug efficacy in females (i.e., only males were tested), lack of additional outcome metrics (e.g., behavior), and that our findings come from a single center, and lack of assessment of cognitive outcome and/or longer-term outcomes [11]. However, the high level of rigor in our study, both technical (randomization and blinding) and statistical, strengthens our overall findings. Additionally, assessing neuroprotective agents across models in a single study is uncommon and has many potential scientific advantages to better predict real-world efficacy and limitations of CNS drugs, as is being demonstrated by the multi-center drug testing consortium for TBI, Operation Brain Trauma Therapy, and/or in consortia in early stages of development for other forms of acute brain injury, including cardiac arrest [39,40,41]. Another limitation was our compound screening design. While logical, it may have incorporated biases that resulted in the elimination of drugs that ultimately warrant testing. For instance, changes in the thresholding rules could have led to other compounds being assessed. Additionally, we did not leverage other sophisticated methods to estimate BBB permeability (e.g., AI learning [42]). Thus, other molecules among the 111 PHLPP inhibitors yet to be characterized could have advantages over NSC74429 (e.g., more neuroprotective, greater ability to penetrate the BBB, and/or less side effects at the optimal dose). Nevertheless, we believe that our approach resulted in exciting new findings that expand the current armamentarium of research tools (NSC117079, NSC45586, NSC74429, and NSC25247) available to researchers to study PHLPPs. Additionally, our findings support the need for further tests on NSC74429 and NSC25247 to modulate PHLPP activity in the brain.

## 5. Patents

Travis C. Jackson and Patrick M. Kochanek are co-inventors on a pending patent application titled: “Compounds for the treatment of acute organ injury” (USPTO Application No. 16/584,314).

## Data Availability

Data is contained within the article.

## Supplementary Materials

The following supporting information can be downloaded at: https://www.mdpi.com/article/10.3390/biom12101352/s1, Figure S1: H&E Staining of CA1 in All Animals in the ACA Study.
