# Supplementary figures and images for: PHLPP Inhibitor NSC74429 Is Neuroprotective in Rodent Models of Cardiac Arrest and Traumatic Brain Injury
Source: Biomolecules. 2022 Sep 23;12(10):1352. doi: 10.3390/biom12101352 (PMC9599532; doi:10.3390/biom12101352)

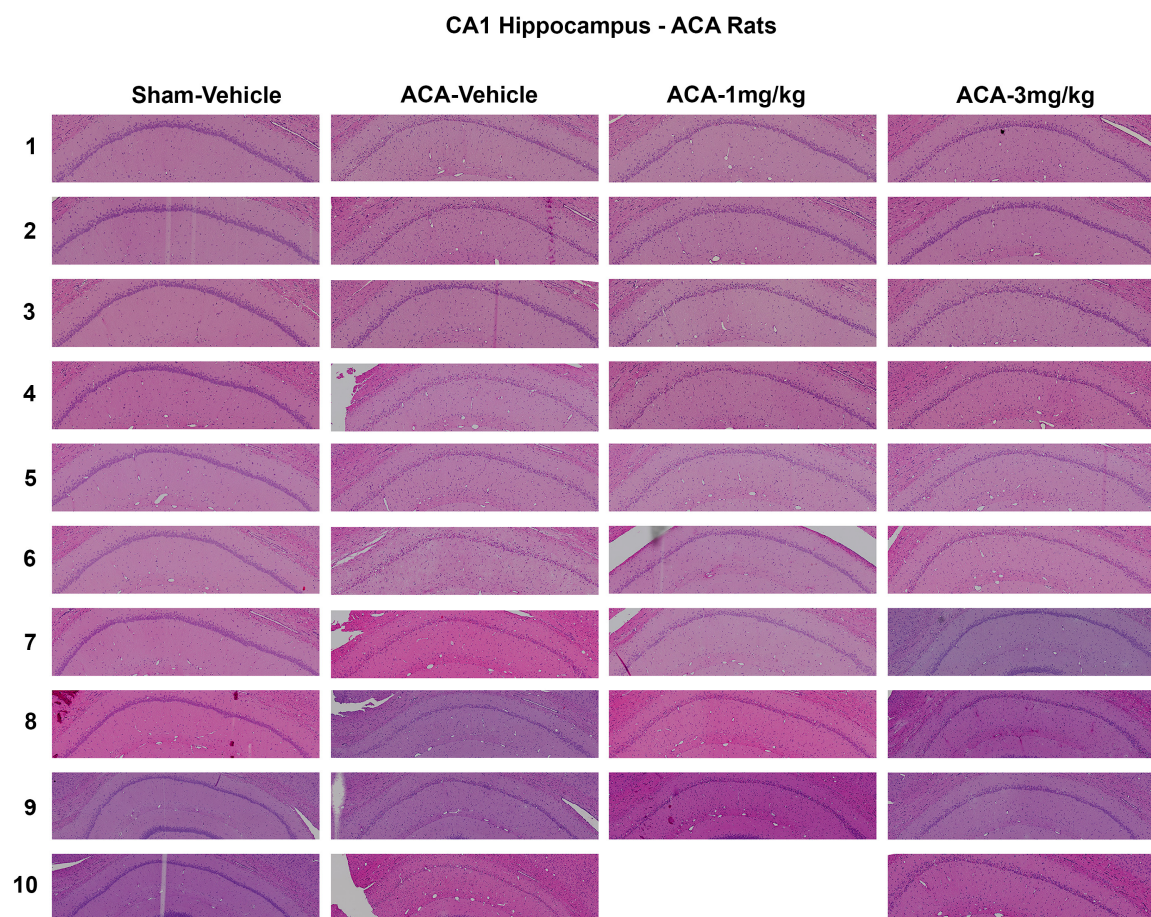

**Figure S1:** H&E Staining of CA1 in All Animals in the ACA Study

Supplement: Supplementary file 1 [file biomolecules-12-01352-s001.zip › biomolecules-1912564-supplementary.pdf]
